# Predictors of rapid eGFR decline in early to moderate chronic kidney disease (stages G1–G4): insights from a real-world Thai cohort incorporating KDIGO 2024 guidelines

**DOI:** 10.1080/0886022X.2025.2593732

**Published:** 2025-11-27

**Authors:** Suthiya Anumas, Poomsit Rattanapanop, Pattharawin Pattharanitima

**Affiliations:** a Chulabhorn International College of Medicine, Thammasat University, Rangsit, Thailand; b Department of Internal Medicine, Thammasat University, Rangsit, Thailand

**Keywords:** Estimated glomerular filtration rate (eGFR), chronic kidney disease (CKD), systolic blood pressure (SBP), statin, albuminuria

## Abstract

Many studies have identified risk factors for kidney failure; however, few have incorporated guideline-based recommendations or evaluated risk across all stages of CKD. This study aimed to identify factors associated with rapid eGFR decline in CKD stages G1–G4 using real-world clinical data. This retrospective cohort study included 2,157 patients with CKD stages 1–4 between January 2018 and December 2022. Rapid eGFR decline was defined as an average eGFR reduction of ≥5 mL/min/1.73 m^2^/year. Clinical variables including age, sex, BMI, diabetes mellitus (DM), hypertension, albuminuria, hemoglobin, systolic blood pressure (SBP), and use of RAASi, GLP-1RA, SGLT2i, and statins were assessed. Multivariable logistic regression was performed to identify independent predictors. Among 2,157 patients, 527 (24.4%) were classified as having rapid eGFR decline. Factors independently associated with rapid decline included SBP ≥120 mmHg (OR 1.84, *p* = 0.001), hemoglobin <10 g/dL (OR 1.78, *p* = 0.002), DM (OR 1.80, *p* < 0.001), and moderately increased albuminuria (OR 1.88, *p* = 0.002); severely increased albuminuria (OR 1.63, *p* = 0.02). In contrast, being in moderate to severe CKD stages (G3–G4) was associated with a lower risk of rapid progression (G3: OR 0.62, *p* = 0.010; G4: OR 0.59, *p* = 0.02). Statin use was also associated with a reduced risk of rapid eGFR decline (OR 0.77, *p* = 0.04). In conclusion, this study identified SBP control, anemia, DM, albuminuria and stage of CKD as key factors associated with rapid CKD progression. Achieving SBP <120 mmHg demonstrated renal benefits. Interestingly, statin use was also associated with a reduced risk of rapid eGFR decline.

## GRAPHICAL ABSTRACT

**Figure:**
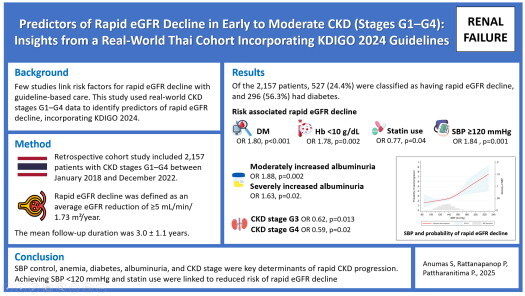

## Introduction

Chronic kidney disease (CKD) is a growing global health burden, affecting an estimated 697.5 million people worldwide, with a prevalence of approximately 9.1% across all stages [1]. In Asia, the prevalence ranges from 7% to 34.3%, accounting for about 434.3 million cases [2]. In Thailand, the estimated prevalence is around 17.5% [3]. The most common underlying causes of CKD are diabetes mellitus (DM) and hypertension (HT), both of which are highly prevalent across affected populations.

Numerous studies have examined the risk factors associated with estimated glomerular filtration rate (eGFR) decline across diverse populations, taking into account variations in race and the underlying causes of CKD. Commonly reported factors include age, sex, and albuminuria, although their effects may vary depending on the study context. In recent years, several clinical practice guidelines for CKD management have been revised, particularly concerning blood pressure targets. The KDIGO 2021^4^ and 2024^5^ have emphasized the role of strict blood pressure control, recommending a target systolic blood pressure (SBP) of <120 mmHg for certain CKD populations. A post-hoc analysis of the SPRINT and ACCORD-BP trials demonstrated that, although an initial reduction in blood pressure in the intensive treatment group led to a transient decline in eGFR, it did not significantly affect the long-term eGFR slope, despite differences in the initial rate of decline [4]. However, real-world data supporting these findings remain limited. Furthermore, emerging evidence supports the use of specific pharmacological therapies, such as renin–angiotensin–aldosterone system inhibitors (RAASi), glucagon-like peptide-1 receptor agonists (GLP-1RA), and sodium–glucose cotransporter-2 inhibitors (SGLT2i), to slow the progression of CKD and reduce mortality in affected patients. These evolving treatment strategies, along with individual patient characteristics, may interact to influence the risk of eGFR decline.

This study aims to identify the risk factors associated with rapid eGFR decline using real-world data. It incorporates traditional predictors such as age, sex, body mass index (BMI), albuminuria level, and CKD stage, while also examining the influence of SBP in accordance with recent clinical guideline updates, along with the impact of recently recommended pharmacological treatments for CKD. The study includes patients across CKD stages G1–G4 to underscore the importance of early risk stratification. By elucidating these associations, the findings may enhance awareness among both patients and healthcare providers that early evaluation and intervention are more effective than delayed management in mitigating CKD progression.

## Methods

### Study population and clinical data

This study included patients with CKD stages G1–G4 during the period from January 1, 2018, to December 31, 2022. A total of 2,157 patients were analyzed. CKD was defined and classified according to the KDIGO guidelines [5,6]. Rapid eGFR decline was defined as an average eGFR reduction of ≥5 mL/min/1.73 m^2^ per year during the follow-up period. In addition, we conducted sensitivity analyses using alternative thresholds of ≥3 and ≥7 mL/min/1.73 m^2^ per year. To minimize variability, the initial eGFR (T1) was calculated as the median of eGFR values measured at the time of study inclusion to 180 days later. Albuminuria levels were categorized according to KDIGO 2024 CKD guidelines [5]. Similarly, the final eGFR (T2) was defined as the median of the last eGFR value to 180 days prior. The rate of eGFR decline was calculated as the difference between T1 and T2, divided by the duration of follow-up. Baseline demographic data, including age, sex, blood pressure, body weight, and comorbidities were obtained from electronic medical records. Laboratory results were recorded as baseline values. The eGFR was calculated using the CKD-EPI 2021 formula. All laboratory tests were performed under accreditation by the Medical Technology Council of Thailand using manufacturer-recommended procedures. CBC was analyzed on a Beckman Coulter system (RBC/PLT by electrical impedance, Hb by spectrophotometry). Serum and urine creatinine were measured by enzymatic method traceable to Isotope Dilution Mass Spectrometry (IDMS), and urine albumin by immunoturbidimetric assay traceable to the International Federation of Clinical Chemistry (IFCC) Certified Reference Material CRM470. Calibration was done with manufacturer-supplied traceable materials, with accuracy verified by internal quality control and external quality assurance. Medication use was defined as having been prescribed a given medication class for at least 180 days during the observational period.

### Study endpoints

The primary endpoint was to identify risk factors associated with rapid eGFR decline. The adjusted risk assessment incorporated traditional variables such as age, sex, body mass index (BMI), and comorbidities including DM and HT, along with the degree of albuminuria and CKD stage. Additionally, key factors of interest included SBP <120 mmHg, as recommended by the KDIGO 2024 guidelines [5], and the use of specific medications, RAASi, GLP-1RA, SGLT2i, and statins.

### Statistical analysis

Continuous variables were reported as mean ± standard deviation (SD) or as median with interquartile range (IQR), depending on the data’s distribution, and were compared using either an unpaired t-test or a Mann–Whitney U test, as applicable. Categorical variables were presented as frequencies and percentages and were analyzed using a Chi-square test or Fisher’s exact test for comparisons.

We addressed missing data in baseline BMI and hemoglobin (Hb) using Multiple Imputation by Chained Equations (MICE) (Table S1). Imputation was performed at baseline (the first observation per participant) using predictive mean matching with *k* = 5 nearest neighbors. Thirty imputed datasets were generated with 10 burn-in iterations, under the missing-at-random assumption. Imputed baseline BMI and Hb values were then carried forward to all follow-up visits as constant covariates, and categorical variables (BMI categories, Hb categories) were derived passively within each imputation. Descriptive statistics (means and SD for continuous variables; proportions for categorical variables) were estimated overall and stratified by rapid progression status. Group differences were evaluated using regression models under the MI framework, providing MI-pooled equivalents of Wilcoxon rank-sum tests for continuous variables and Fisher’s exact or χ^2^ tests for categorical variables.

To evaluate eGFR changes during the follow-up period and their association with baseline factors, we used mixed-effects model analysis, with eGFR at each time point as the repeated outcome and baseline covariates as fixed predictors.

To identify factors independently associated with rapid eGFR decline, multivariable logistic regression analysis was performed. Variables included in the model were age, sex, BMI, DM, HT, CKD stage, albuminuria level, SBP <120 mmHg, and the use of RAASi, GLP-1RA, SGLT2i, and statins. Odds ratios (ORs) with 95% confidence intervals (CIs) were reported. We also conducted secondary analyses by modeling continuous SBP with restricted cubic splines to examine its association with the probability of rapid eGFR decline. All statistical tests were two-sided, and a p-value <0.05 was considered statistically significant. Analyses were conducted using Stata version 17.0 BE (StataCorp, College Station, TX, USA).

## Results

**Figure 1.**
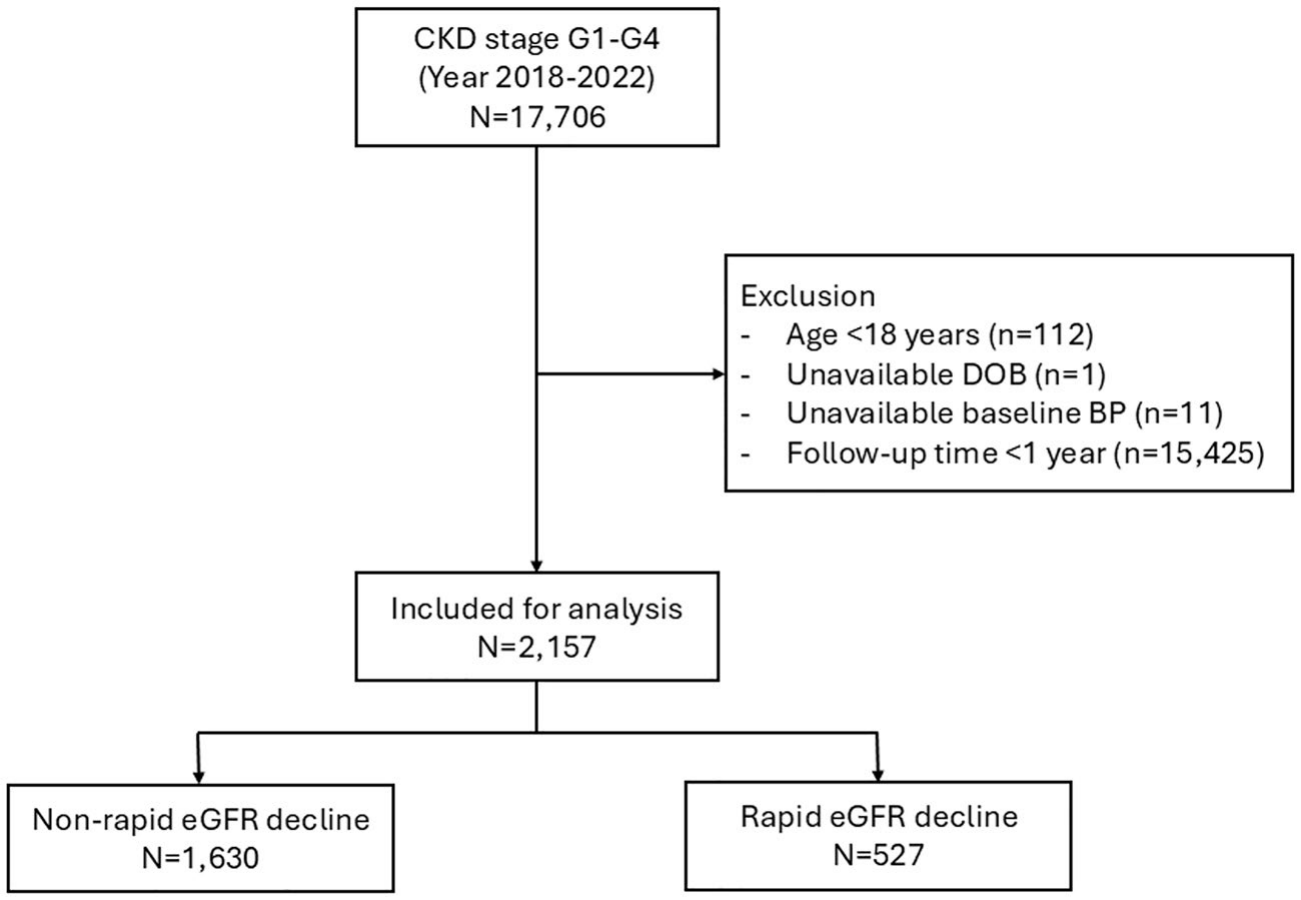
Study flow. Abbreviations: BP, blood pressure; CKD, chronic kidney disease; DOB, date of birth

A total of 2,157 patients with CKD stages G1–G4 were included in the analysis. Among these, 527 patients (24.4%) were identified as rapid eGFR decline, defined by an annual eGFR decline of ≥5 mL/min/1.73 m^2^/year. The mean follow-up duration was 3.0 ± 1.1 years (Figure 1).

### Baseline characteristics

Compared with non-rapid eGFR decline, rapid eGFR decline had a significantly lower mean hemoglobin level (11.9 vs. 12.2 g/dL, *p* < 0.001), higher SBP (137.6 vs. 133.4 mmHg, *p* < 0.001), and faster rate of eGFR decline (mean −8.4 vs. −0.9 mL/min/1.73 m^2^/year, *p* < 0.001). Rapid eGFR decline also had a significantly higher prevalence of hypertension (64.0% vs. 58.0%, *p* = 0.02) and diabetes (56.2% vs. 38.5%, *p* < 0.001). Albuminuria levels were also significantly higher among rapid eGFR decline, with severely increased albuminuria observed in 10.3% of rapid eGFR decline vs. 1.8% in non-rapid eGFR decline (*p* < 0.001). The majority of patients in this cohort were in the early to moderate stages of CKD (G1–G2) rather than advanced stages (G3–G4), accounting for 63.8% and 36.2%, respectively.

Regarding medication use, rapid eGFR decline were more likely to receive RAASi (67.6% vs. 58.2%, *p* < 0.001), GLP-1RA (8.2% vs. 5.3%, *p* = 0.02), and SGLT2i (19.0% vs. 12.6%, *p* < 0.001). However, statin use was slightly less common among rapid eGFR decline (54.3% vs. 53.6%, *p* = 0.76) (Table 1).

**Table 1.** Baseline characteristic.

Characteristics	Non-rapid eGFR decline (*n* = 1,630)	Rapid eGFR decline (*n* = 527)	Total (*n* = 2,157)	p-value
Age, years, mean (SD)	59.1 (17.1)	59.2 (15.5)	59.1 (16.7)	0.64
Female, n (%)	934 (57.3)	286 (54.3)	1,220 (56.6)	0.23
Comorbidities, n (%)				
CAD	120 (7.4)	37 (7.0)	157 (7.3)	0.85
CVD	31 (1.9)	12 (2.3)	43 (2.0)	0.60
HT	945 (58.0)	337 (64.0)	1,282 (59.4)	0.02
DM	627 (38.5)	296 (56.2)	923 (42.8)	<0.001
DLP	836 (51.3)	294 (55.8)	1,130 (52.4)	0.08
SBP, mmHg, mean (SD)	133.4 (15.7)	137.6 (17.1)	134.4 (16.1)	<0.001
DBP, mmHg, mean (SD)	77.2 (10.5)	78.0 (10.7)	77.4 (10.5)	0.18
BMI, kg/m^2^, mean (SD)	25.3 (5.1)	25.6 (5.7)	25.5 (5.4)	<0.001
Rate of eGFR decline (mL/min/1.73 m^2^/y), median (IQR) (T2-T1)	−0.9 (−2.3 to 0.9)	−8.4 (−13.0 to −6.5)	−1.80 (−4.9 to 0.3)	<0.001
Baseline eGFR, mL/min/1.73 m^2^, mean (SD)	73.8 (32.1)	75.9 (30.2)	74.3 (31.6)	0.22
CKD stage				0.04
eGFR ≥90 mL/min/1.73 m^2^	594 (36.4)	204 (38.7)	798 (37.0)	
eGFR ≥60 to <90 mL/min/1.73 m^2^	420 (25.8)	158 (30.0)	578 (26.8)	
eGFR ≥30 to <60 mL/min/1.73 m^2^	443 (27.2)	122 (23.2)	565 (26.2)	
eGFR ≥15 to <30 mL/min/1.73 m^2^	173 (10.6)	43 (8.2)	216 (10.0)	
Laboratory				
Hb, g/dL, mean (SD)	12.2 (1.9)	11.9 (2.0)	11.8 (2.0)	<0.001
Cholesterol, mg/dL, mean (SD)	183.5 (52.9)	180.1 (57.8)	182.6 (54.3)	0.17
TG, mg/dL, mean (SD)	137.1 (88.2)	144.0 (83.8)	138.9 (87.1)	0.053
LDL, mg/dL, mean (SD)	102.5 (39.4)	103.4 (42.0)	102.7 (40.0)	0.98
HDL, mg/dL, mean (SD)	51.8 (16.4)	51.6 (17.3)	51.7 (16.6)	0.25
UACR				<0.001
Normal to mildly increased albuminuria	1,514 (92.9)	406 (77.0)	1,920 (89.0)	
Moderately increased albuminuria	86 (5.3)	67 (12.7)	153 (7.1)	
Severely increases albuminuria	30 (1.8)	54 (10.3)	84 (3.9)	
Medication, n (%)				
RAASi	950 (58.3)	356 (67.6)	1306 (60.6)	<0.001
GLP-1RA	86 (5.3)	43 (8.2)	129 (6.0)	0.02
SGLT2i	205 (12.6)	100 (19.0)	305 (14.1)	<0.001
Statin	871 (53.4)	286 (54.3)	1,157 (53.6)	0.76

Abbreviations: BMI, body mass index; CAD, coronary artery disease; CKD, chronic kidney disease; CVD, cerebrovascular disease; DBP, diastolic blood pressure; DLP, dyslipidemia; DM, diabetes mellitus; eGFR, estimated glomerular filtration rate; GLP-1RA, glucagon like-peptide receptor agonist; Hb, hemoglobin; HDL, high density lipoprotein; HT, hypertension; LDL, low-density lipoprotein; RAASi, renin-angiotensin-aldosterone system; SBP, systolic blood pressure; SGLT2i, sodium–glucose cotransporter-2 inhibitor; TG, triglyceride; UACR, urine albumin creatinine ratio.

Among patients with rapid eGFR decline, the percentage of eGFR loss from baseline decreased by approximately 10% every 6–12 months, reaching about 50% loss of baseline eGFR by the end of follow-up. In contrast, non-rapid progressors maintained relatively stable kidney function with only minimal decline (Figure S1).

### Mixed-effect model

In the mixed-effects model with random intercept and slope, eGFR declined significantly over time in the total cohort (β = −0.008 per day, 95% CI −0.009 to −0.007; *p* < 0.001), corresponding to an average annual loss of approximately 2.9 mL/min/1.73 m^2^. SBP ≥120 mmHg was associated with lower eGFR throughout the follow-up period (β = −2.98, 95% CI −4.44 to −1.56; *p* < 0.001). Older age (>60 years), male sex, hypertension, and severe albuminuria were also significantly associated with lower eGFR values. In addition, statin use was associated with lower eGFR during follow-up (β = −1.15, 95% CI −2.21 to −0.09; *p* = 0.035), an association that likely reflects underlying comorbidity rather than a direct pharmacological effect (Table S2).

### Factors associated with rapid eGFR decline

**Figure 2.**
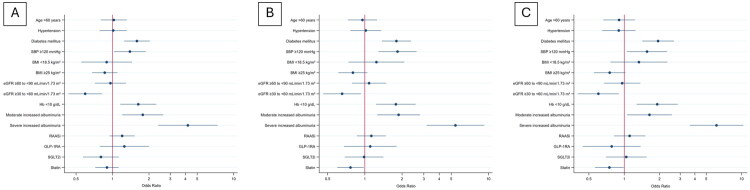
Adjusted HRs for risk of rapid eGFR decline. Definition of rapid eGFR decline is (A) ≥3 mL/min/1.73 m^2^/year, (B) ≥5 mL/min/1.73 m^2^/year, and (C) ≥7 mL/min/1.73 m^2^/year. Abbreviations: BMI, body mass index; eGFR, estimated glomerular filtration rate; GLP-1RA, glucagon-like peptide-1 receptor agonist; RAASi, renin-angiotensin-aldosterone system; SBP, systolic blood pressure; SGLT2i, sodium–glucose cotransporter-2 inhibitor

We then examined factors associated *with* rapid eGFR decline using logistic regression, defining rapid decline as an annual loss of ≥5 mL/min/1.73 m^2^/year. In the multivariable logistic regression analysis, several factors were independently associated with rapid eGFR decline. These included SBP ≥120 mmHg (OR 1.84, 95% CI 1.29–2.61, *p* = 0.001), hemoglobin <10 g/dL (OR 1.78, 95% CI 1.24–2.57, *p* = 0.002), diabetes mellitus (OR 1.80, 95% CI 1.37–2.36, *p* < 0.001), and elevated albuminuria levels, with moderately increased albuminuria (OR 1.88, 95% CI 1.26–2.79, *p* = 0.002) and severely increased albuminuria (OR 1.63, 95% CI 1.08–3.16, *p* = 0.02). In contrast, statin use was associated with a reduced risk of rapid eGFR decline (OR 0.77, 95% CI 0.59–0.99, *p* = 0.04). Patients with moderate CKD stages (G3 and G4) also showed a lower risk compared to those with preserved kidney function (G1), with ORs of 0.62 (95% CI 0.47–0.91, *p* = 0.01) and 0.59 (95% CI 0.37–0.92, *p* = 0.02), respectively. Other variables, including age, sex, BMI, hypertension, and use of RAASi, GLP-1RAs, or SGLT2i, were not significantly associated with rapid progression after adjustment (Table 2 and Figure 2).

**Table 2.** Risk association of rapid eGFR decline.

Risks	Univariable OR	95%CI	p-value	Multivariable OR	95% CI	p-value
Age >60 years	0.95	0.78–1.15	0.59	0.96	0.73–1.26	0.75
Female	0.88	0.73–1.08	0.22	0.87	0.68–1.19	0.25
Hypertension	1.29	1.05–1.58	0.02	1.02	0.76–1.36	0.91
Diabetes mellitus	2.05	1.68–2.50	<0.001	1.80	1.38–2.36	<0.001
SBP ≥120 mmHg	1.65	1.25–2.19	<0.001	1.84	1.29–2.61	0.001
BMI <18.5 Kg/m^2^	1.16	0.72–1.87	0.55	1.24	0.74–2.09	0.41
BMI ≥25 Kg/m^2^	0.87	0.69–1.09	0.23	0.82	0.61–1.05	0.11
eGFR ≥90 mL/min/1.73 m^2^	Reference			Reference		
eGFR ≥60 to <90 mL/min/1.73 m^2^	1.10	0.86–1.40	0.46	1.80	0.79–1.49	0.63
eGFR ≥30 to <60 mL/min/1.73 m^2^	0.80	0.62–1.04	0.09	0.62	0.47– 0.91	0.01
eGFR ≥15 to <30 mL/min/1.73 m^2^	0.72	0.50–1.05	0.09	0.59	0.37–0.92	0.02
Hb <10 g/dL	1.44	1.05–1.98	0.02	1.78	1.24–2.57	0.002
Normal to mildly increased albuminuria	Reference			Reference		
Moderately increased albuminuria	2.91	2.07–4.07	<0.001	1.88	1.26–2.79	0.002
Severely increased albuminuria	6.71	4.24–10.63	<0.001	1.63	1.08–3.16	0.02
RAASi	1.49	1.21–1.83	<0.001	1.13	0.86–1.48	0.38
GLP-1RA	1.60	1.09–2.33	0.02	1.17	0.68–1.53	0.44
SGLT2i	1.63	1.25–2.12	<0.001	0.99	0.69–1.41	0.94
Statin	1.03	0.85–1.26	0.74	0.77	0.59–0.99	0.04

Abbreviations: BMI, body mass index; CKD, chronic kidney disease; DM, diabetes mellitus; eGFR, estimated glomerular filtration rate; GLP-1RA, glucagon-like peptide-1 receptor agonist; Hb, hemoglobin; HT, hypertension; RAASi, renin-angiotensin-aldosterone system; SBP, systolic blood pressure; SGLT2i, sodium–glucose cotransporter-2 inhibitor.

### Sensitivity analysis

#### *Definition of rapid eGFR decline:* ≥*3 mL/min/1.73 m^2^/year*

In multiple logistic regression, factors significantly associated with rapid eGFR decline included systolic blood pressure ≥120 mmHg (OR 1.40, 95% CI 1.03–1.88, *p* = 0.03), hemoglobin <10 g/dL (OR 1.63, 95% CI 1.16–2.30, *p* = 0.004), diabetes mellitus (OR 1.59, 95% CI 1.25–2.04, *p* < 0.001), moderately increased albuminuria (OR 1.78, 95% CI 1.08–2.62, *p* = 0.02), and severely increased albuminuria (OR 4.20, 95% CI 2.38–9.27, *p* < 0.001). Compared with participants with preserved kidney function (G1), those with CKD stage G3 had a lower risk of rapid progression (OR 0.75, 95% CI 0.46–0.99, *p* = 0.04). Other variables, including age, sex, BMI, hypertension, and use of RAAS inhibitors, GLP-1 receptor agonists, SGLT2 inhibitors, and statins, were not significantly associated with rapid progression after adjustment (Figure 2).

#### *Definition of rapid eGFR decline:* ≥*7 mL/min/1.73 m^2^/year*

Multivariable logistic regression identified significant risk associations for rapid eGFR decline with systolic blood pressure ≥120 mmHg (OR 1.56, 95% CI 1.09–2.32, *p* = 0.02), hemoglobin <10 g/dL (OR 1.91, 95% CI 1.28–2.87, *p* = 0.002), diabetes mellitus (OR 1.94, 95% CI 1.43–2.60, *p* < 0.001), and moderately increased albuminuria (OR 1.61, 95% CI 1.06–2.46, *p* = 0.03). In contrast, compared with CKD stage G1, both stage G3 (OR 0.62, 95% CI 0.48–0.90, *p* = 0.009) and stage G4 (OR 0.43, 95% CI 0.24–0.74, *p* = 0.003) were associated with a lower risk of rapid decline. Statin therapy was also associated with reduced risk (OR 0.74, 95% CI 0.57–0.99, *p* = 0.04). Other variables including age, sex, BMI, hypertension, and use of RAAS inhibitors, GLP-1 receptor agonists, and SGLT2 inhibitors showed no significant association after adjustment (Figure 2).

### Secondary analysis: continuous SBP and risk of rapid eGFR decline

**Figure 3.**
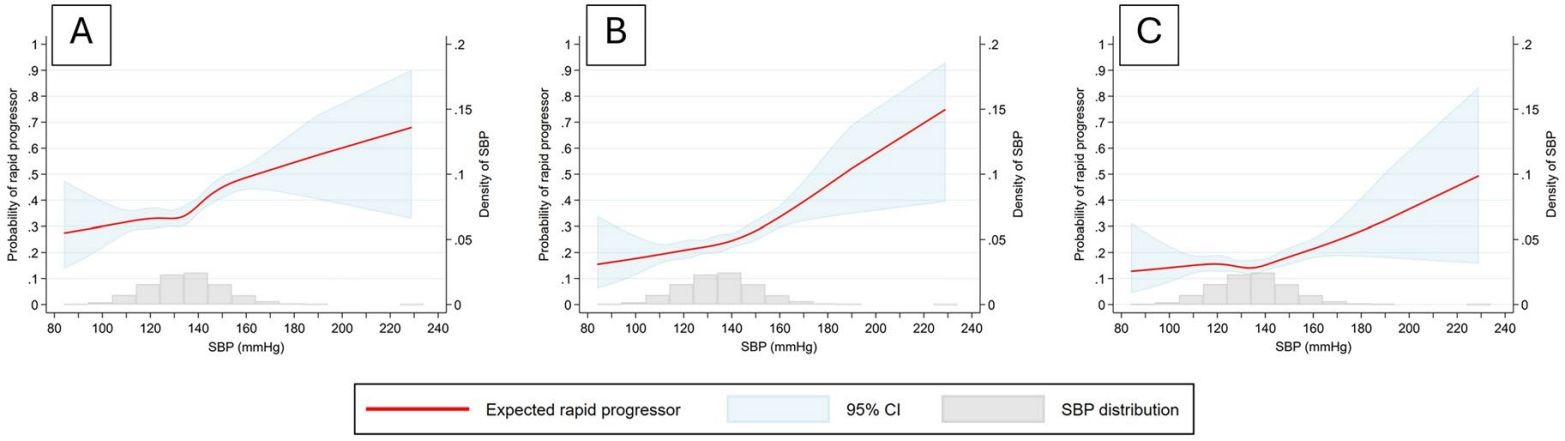
Association between systolic blood pressure (SBP) and the probability of rapid eGFR decline at different thresholds (≥3, ≥5, and ≥7 mL/min/1.73 m^2^/year). Predicted probabilities were estimated using restricted cubic splines. The red line represents the expected probability of rapid progression, the shaded blue area the 95% confidence interval, and the gray histogram the distribution of SBP in the study population. Definition of rapid eGFR decline is A) ≥3 mL/min/1.73 m^2^/year, B) ≥5 mL/min/1.73 m^2^/year, and C) ≥7 mL/min/1.73 m^2^/year.

**Figure 4.**
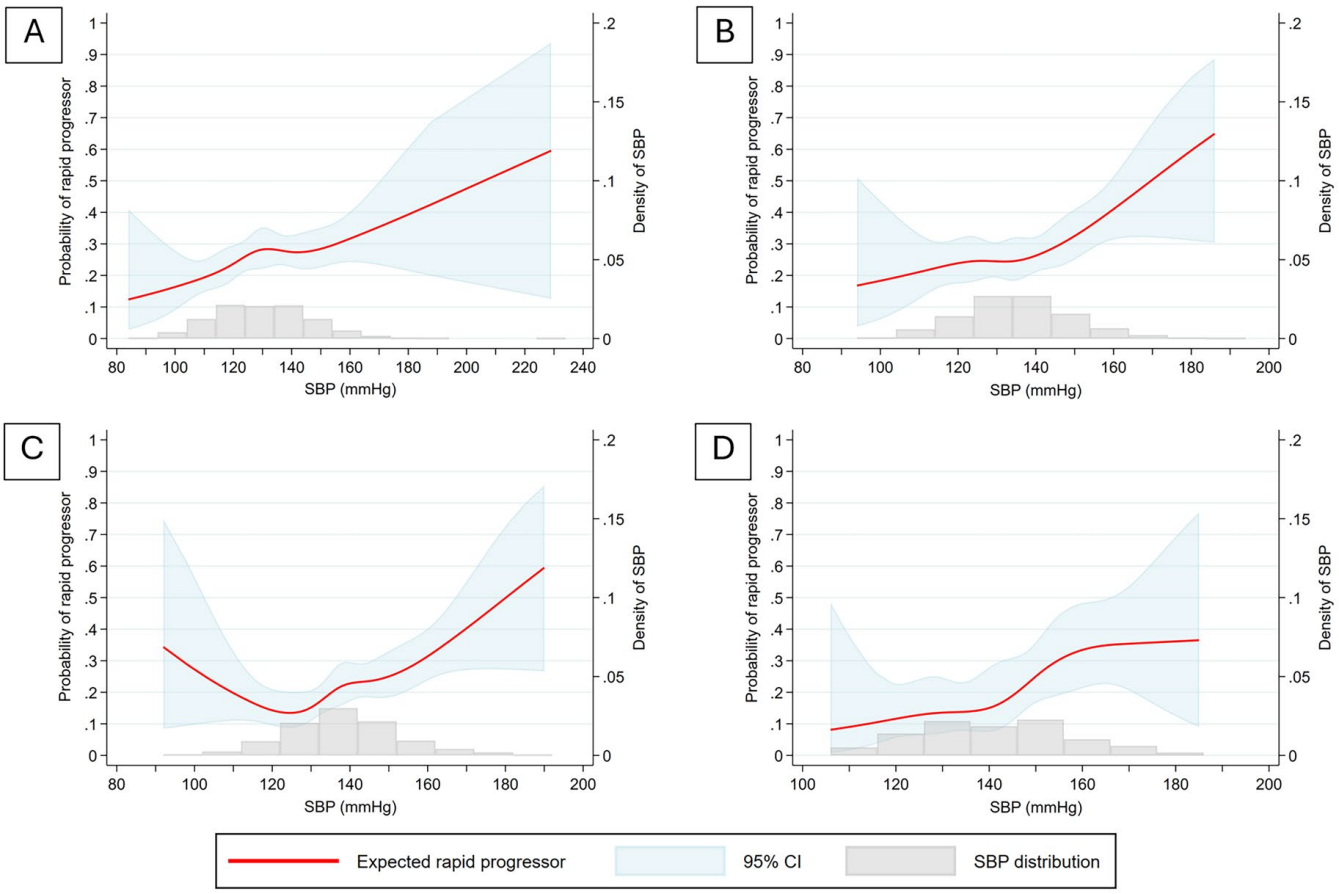
Association between systolic blood pressure (SBP) and the probability of rapid eGFR decline ≥5 mL/min/1.73 m^2^/year stratified by CKD stage G1–G4 (A–D). Predicted probabilities were estimated using restricted cubic splines. The red line represents the expected probability of rapid progression, the shaded blue area the 95% confidence interval, and the gray histogram the distribution of SBP in the study population.

Using restricted cubic splines, higher SBP was consistently associated with a greater probability of rapid eGFR decline across all definitions (≥3, ≥5, and ≥7 mL/min/1.73 m^2^/year) (Figure 3). The probability of rapid progression increased in an approximately linear fashion above 120 mmHg. When stratified by CKD stage (G1–G4) using the ≥5 mL/min/1.73 m^2^ definition, higher SBP was generally associated with a greater risk of rapid eGFR decline, although the shape of the association varied. In G1 and G2, the probability of rapid progression rose progressively above 120 mmHg, whereas in G3 and G4 the curves were flatter at lower SBP but increased more steeply at higher levels (Figure 4).

## Discussion

In this real-world cohort of 2,157 patients with CKD stages G1–G4, approximately 24.4% were identified as rapid eGFR decline, defined by an annual eGFR decline of ≥5 mL/min/1.73 m^2^. Our analysis revealed that several clinical factors, including SBP ≥120 mmHg, hemoglobin <10 g/dL, higher levels of albuminuria, and diabetes mellitus, were independently associated with rapid eGFR decline. Conversely, the use of statins and being in moderate to severe CKD stages (G3–G4) were associated with a lower risk of rapid progression.

Our study included patients with CKD stages G1–G4, allowing us to evaluate risk factors for rapid eGFR decline across the full spectrum of early to advanced non-dialysis CKD. Although patients with CKD stages G1–G2 may not yet experience the complications typically associated with morbidity and mortality, including these stages is essential to promote early awareness, monitoring, and understanding of disease progression in all CKD patients. In contrast, we excluded patients with CKD stage G5, as identifying rapid eGFR decline at this stage provides limited clinical benefit; by then, most patients are already approaching or undergoing kidney replacement therapy. Most previous studies have used outcomes such as initiation of dialysis, kidney transplantation, or a 30–50% decline in eGFR, which are highly relevant for patients with moderate to severe CKD. However, in our study, we selected rapid eGFR decline as the primary outcome, as we believe this marker is more suitable for assessing disease progression across all CKD stages, including early and moderate stages where more proactive interventions are possible. To minimize confounding from baseline renal function, CKD stage was included as an adjusted covariate in our multivariable analysis, allowing us to better isolate the effects of other risk factors independent of CKD severity.

The identified risk factors, low hemoglobin, elevated albuminuria levels, and diabetes mellitus, are consistent with findings from numerous previous studies [7–10]. The underlying pathophysiology of albuminuria and diabetes is closely linked to chronic inflammation, endothelial dysfunction, and progressive renal fibrosis, all of which contribute to accelerated kidney damage [11,12]. Low hemoglobin has also been widely reported as a predictor of CKD progression, likely due to its contribution to renal tissue hypoxia. Inadequate oxygen delivery may exacerbate tubular injury, promote interstitial fibrosis, and accelerate functional decline [13,14]. Collectively, these mechanisms help elucidate the strong association observed between these clinical factors and rapid eGFR decline in our cohort.

For CKD stages G3 and G4, the observed association with a lower risk of rapid eGFR decline may be partially explained by the fact that, at these stages, changes in serum creatinine have a smaller impact on eGFR compared to earlier stages of CKD. For example, in a 40-year-old male, an increase in serum creatinine from 1.2 to 1.3 mg/dL results in a decrease in eGFR from 78.4 to 71.2 mL/min/1.73 m^2^, whereas an increase from 1.6 to 1.7 mg/dL leads to a change in eGFR from 55.5 to 51.6 mL/min/1.73 m^2^. This illustrates that the same absolute increase in serum creatinine leads to a smaller change in eGFR in more advanced CKD stages.

Studies have reported that a one-year deceleration in eGFR decline is associated with a 20% reduction in the risk of subsequent kidney failure requiring replacement therapy (KFRT). However, this deceleration appears to be less pronounced in patients with more advanced stages of kidney disease [15]. These findings suggest that in advanced CKD, even when the rate of eGFR decline does not meet the threshold for rapid progression, it may still significantly impact clinical outcomes. Therefore, the clinical application of eGFR change interpretation should be considered within this context.

In recent years, the KDIGO 2021 Blood Pressure Guideline [16] and KDIGO 2024 CKD Guideline [5] have recommended a target SBP <120 mmHg for patients with CKD, based largely on findings from the SPRINT trial. The SPRINT trial (2015) [17] demonstrated that intensive SBP control significantly reduced cardiovascular events and all-cause mortality. However, it was also associated with an increased risk of acute kidney injury (AKI) and an initial decline in renal function among participants without CKD. In the extended follow-up phase, with a median duration of 3.33 years, comparable to our study, no significant long-term adverse effects on renal outcomes were observed [18]. A *post hoc* analysis of the SPRINT and ACCORD-BP trials concluded that there was no significant difference in the long-term eGFR slope based on the initial eGFR decline or blood pressure target, regardless of whether patients received intensive or standard treatment [4]. In contrast, our real-world cohort study found that patients who maintained SBP <120 mmHg at baseline had a significantly lower risk of rapid eGFR decline, whether defined as ≥5 mL/min/1.73 m^2^/year or in sensitivity analyses using thresholds of ≥3 or ≥7 mL/min/1.73 m^2^/year. Furthermore, secondary analyses demonstrated that the association between higher SBP and the probability of rapid eGFR decline was consistent across CKD stages. These findings suggest that, outside of randomized controlled trial settings, tighter SBP control may be associated with renal protection. Supporting this, a Thai national database study demonstrated that SBP ≥120 mmHg was associated with accelerated kidney function loss in diabetic patients [9], and a Chinese CKD cohort found that elevated SBP was linked to *a* ≥ 50% decline in eGFR among patients with CKD stages G2–G5ND^9^. However, as our study is based on a retrospective cohort design, we cannot establish a causal relationship between SBP levels and eGFR decline. Nonetheless, given that the SPRINT trial did not show harm from intensive SBP control and our study observed a potential renal benefit, these findings collectively reinforce the importance of strict blood pressure control in CKD patients and support the updated KDIGO 2024 CKD guideline [5] recommendation to target SBP <120 mmHg when clinically appropriate.

Interestingly, our study demonstrated that statin use was independently associated with a lower risk of rapid eGFR decline, suggesting a potential renoprotective effect. Although the mixed-effects model showed that statin use was associated with lower eGFR values during the follow-up period—likely reflecting comorbidities that serve as indications for statin therapy—the logistic regression model indicated that statin use was associated with a lower risk of rapid eGFR decline. This observation is supported by findings from previous clinical studies that have proposed beneficial renal effects of statins. A meta-analysis reported a reduction in urinary albumin excretion with statin therapy, although it did not show a significant impact on the rate of eGFR decline [19]. Similarly, the PLANET I study observed modest reductions in proteinuria and a slower decline in eGFR [20], further supporting the hypothesis of statins’ renal benefits. These effects are thought to arise from the pleiotropic properties of statins, including their anti-inflammatory, antioxidant, and immunomodulatory actions, as well as their ability to mitigate lipotoxicity, which is associated with key pathological mechanisms underlying CKD progression [21,22]. However, future prospective studies are needed to confirm the causal relationship between statin use and CKD progression, and such studies should also explore changes in albuminuria and inflammatory markers. Unfortunately, in our cohort, RAASi, GLP-1RA, and SGLT2i did not show a statistically significant association with reduced risk of rapid eGFR decline. The lack of observed benefit for GLP-1RA and SGLT2i may be attributed to the limited proportion of patients receiving these medications, due to reimbursement restrictions in Thailand, which could have reduced the statistical power to detect meaningful effects. This reflects a broader challenge in many low to middle income countries, where access to proven renoprotective therapies remains restricted. Expanding affordability and accessibility should be prioritized in health policy to reduce CKD progression and its societal burden. As for RAASi, their renoprotective benefits are well-established, particularly in patients with moderate to severe albuminuria [23,24]. However, in our study population, a relatively small number of patients had moderate to severe levels of albuminuria, which may explain the absence of a detectable benefit from RAASi use in this analysis.

In sensitivity analyses using different thresholds to define rapid eGFR decline, the associations remained consistent: diabetes, SBP ≥120 mmHg, hemoglobin <10 g/dL, and albuminuria were linked to higher risk of decline. These findings have important implications for regional health systems. Patients with DM, elevated SBP, anemia, or albuminuria may require closer surveillance with more frequent eGFR testing and routine monitoring, enabling earlier identification of rapid progressors and targeted interventions, which may help mitigate CKD progression despite restricted access to novel therapies.

Our study has several notable strengths. First, we included patients across all stages of CKD (stage G1-G4), allowing for broader applicability of our findings to a wide range of clinical scenarios. Second, we utilized real-world data, and all the risk factors assessed, such as blood pressure, hemoglobin levels, and medication use, are routinely available in everyday clinical practice. Third, our analysis incorporated recent recommendations from the KDIGO 2024 guidelines [5] regarding blood pressure and hemoglobin targets, as well as commonly prescribed medications in CKD management. This approach enhances the clinical relevance of our findings. However, several limitations must be acknowledged. First, due to the retrospective nature of the study design, we are unable to establish causal relationships. Our findings reflect associations rather than direct effects. Second, medication exposure was defined pragmatically, and we could not apply new-user designs or causal inference methods because initiation dates and dosing data were unavailable. Third, the study was conducted at a single center, which may limit the generalizability of the results to broader CKD populations or other healthcare settings. Lastly, the factors used in our analysis were measured only at baseline, with the exception of eGFR, which may not fully capture dynamic changes over time. Future studies should be conducted in multicenter settings or in larger populations of the same ethnicity to strengthen the robustness of the outcomes.

## Conclusion

Our findings emphasize the importance of controlling SBP, managing albuminuria, correcting anemia, and possibly leveraging the protective effects of statins to mitigate rapid CKD progression. These results support recent KDIGO 2024 guideline recommendations and underscore the need for early identification and personalized intervention in patients at high risk of kidney function decline.

## Supplementary Material

Supplement R1.docx

## Data Availability

The cleaned and deidentified dataset supporting the findings of this study has been deposited in a public GitHub repository and can be accessed at https://github.com/CKDprogression/Predictors-of-Rapid-eGFR-Decline-in-CKD.git. All data have been fully anonymized in accordance with ethical guidelines and regulatory requirements.
