# Supplementary material for: Predictors of rapid eGFR decline in early to moderate chronic kidney disease (stages G1–G4): insights from a real-world Thai cohort incorporating KDIGO 2024 guidelines
Source: Ren Fail. 2025 Nov 27;47(1):2593732. doi: 10.1080/0886022X.2025.2593732 (PMC12667306; doi:10.1080/0886022X.2025.2593732)
Supplement: Supplement R1.docx [file IRNF_A_2593732_SM6814.docx]

**Supplementary document**

**Table S1. Summary of missing data for key study variables**

| **Variable** | **Number of missing values** | **% Missing values** |
| --- | --- | --- |
| Hemoglobin | 381 | 21.5 |
| BMI | 488 | 29.2 |

**Table S2. Mixed-effects model of baseline factors associated with eGFR over time.**

| **Variable** | **Model 1: Random intercept and slope (no interaction)** | |
| --- | --- | --- |
|  | **β (95% CI)** | **p-value** |
| Time (days) | −0.008 (−0.009, −0.007) | <0.001 |
| SBP ≥120 mmHg | −2.98 (−4.44, −1.56) | <0.001 |
| Time × SBP ≥120 mmHg | — | — |
| Age >60 years | −5.78 (−6.98, −4.59) | <0.001 |
| Female sex | 1.65 (0.58, 2.73) | 0.003 |
| Hypertension | −2.24 (−3.48, −1.00) | <0.001 |
| Diabetes mellitus | −0.85 (−2.04, 0.33) | 0.157 |
| BMI <18.5 Kg/m^2^ | 0.51 (−1.77, 2.79) | 0.663 |
| BMI ≥25 Kg/m^2^ | 1.15 (−0.99, 3.30) | 0.295 |
| CKD stage G2 | −28.48 (−29.92, −27.04) | <0.001 |
| CKD stage G3 | −56.88 (−58.63, −55.14) | <0.001 |
| CKD stage G4 | −88.23 (−90.27, −86.22) | <0.001 |
| Hb <10 g/dL | −1.07 (−2.69, 0.56) | 0.199 |
| Moderately increased albuminuria | −0.65 (−2.59, 1.28) | 0.508 |
| Severely increased albuminuria | −3.30 (−5.77, −0.82) | 0.01 |
| RAASi use | 0.70 (−0.45, 1.86) | 0.233 |
| GLP1RA use | −0.90 (−3.14, 1.35) | 0.435 |
| SGLT2i use | 1.30 (−0.35, 2.95) | 0.121 |
| Statin use | −1.15 (−2.21, −0.09) | 0.035 |

**Abbreviation:** BMI, body mass index; CKD, chronic kidney disease; GLP-1RA, glucagon like-peptide receptor agonist; Hb, hemoglobin; RAASi, renin-angiotensin-aldosterone system; SBP, systolic blood pressure; SGLT2i, sodium–glucose cotransporter-2 inhibitor


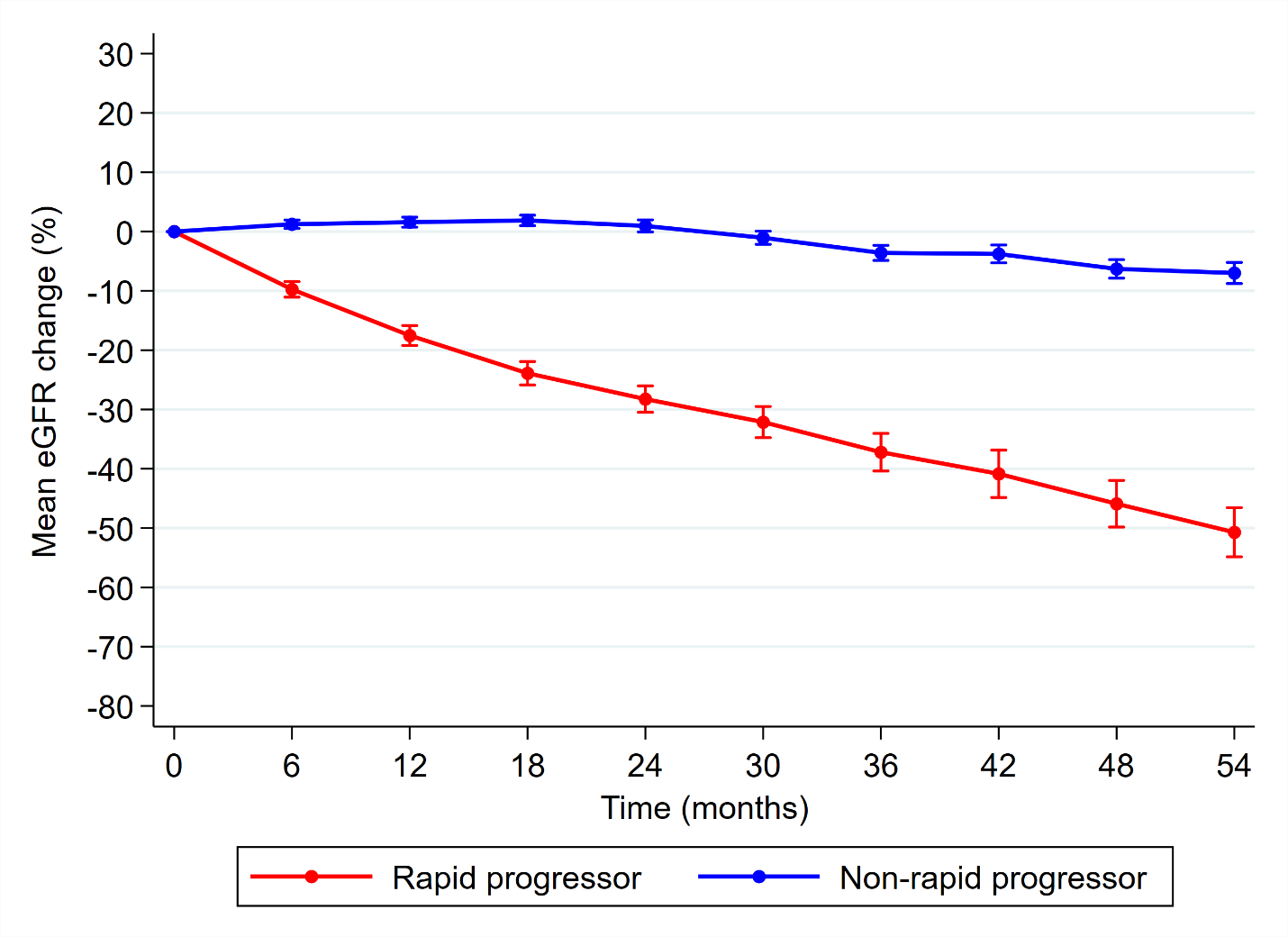


**Figure S1. Longitudinal change in mean eGFR among rapid and non-rapid progressors.**
The figure shows the mean percentage change in eGFR from baseline over 54 months of follow-up. Patients classified as rapid progressors (red line) demonstrated a steady and marked decline in kidney function, with approximately 50% loss of baseline eGFR by the end of follow-up. In contrast, non-rapid progressors (blue line) maintained relatively stable kidney function, with only minimal decline. Error bars represent 95% confidence intervals.
